# Integrated DNA methylation and gene expression analysis identifies SLAMF7 as a key regulator of atherosclerosis

**DOI:** 10.18632/aging.101470

**Published:** 2018-06-13

**Authors:** Zhangyong Xia, Mingliang Gu, Xiaodong Jia, Xiaoting Wang, Chunxia Wu, Jiangwen Guo, Liyong Zhang, Yifeng Du, Jiyue Wang

**Affiliations:** 1 Department of Neurology, Liaocheng People's Hospital and Liaocheng Clinical School of Taishan Medical University, Liaocheng, Shandong 252000, P.R. China; 2 Joint Laboratory for Translational Medicine Research, Beijing Institute of Genomics, Chinese Academy of Sciences & Liaocheng People's Hospital, CAS Key Laboratory of Genomic Science and Information, Chinese Academy of Sciences, Beijing 100101, P.R. China; 3 Joint Laboratory for Translational Medicine Research, Beijing Institute of Genomics, Chinese Academy of Sciences and Liaocheng People's Hospital, Liaocheng, Shandong 252000, P.R. China; 4 Taishan Medical University, Taian, Shandong 271016, P.R. China; 5 Department of Ultrasonic, Liaocheng People's Hospital and Liaocheng Clinical School of Taishan Medical University, Liaocheng, Shandong 252000, P.R. China; 6 Deparment of Neurology, Second Affiliated Hospital of Guangzhou University of Traditional Chinese Medicine, Guangzhou, Guangdong 510120, P.R. China; 7 Department of Neurosurgery, Liaocheng People’s Hospital and Liaocheng Clinical School of Taishan Medical University, Liaocheng, Shandong 252000, PR China; 8 Department of Neurology, Shandong Provincial Hospital Affiliated to Shandong University, Jinan, Shandong 250021, P.R. China

**Keywords:** atherosclerosis, DNA methylation, gene expression, SLAMF7

## Abstract

Atherosclerosis (AS) is a multifactorial disease. Exploration of DNA methylation in regulating gene transcription in a cell type- and stage-specific manner will shed light on understanding the biological processes associated with plaque stability. We identified 174 up-regulated genes with hypo-methylation in the promoter, and 86 down-regulated genes with hyper-methylation in the promoter, in AS vs. healthy controls. Among them, high expression of signaling lymphocytic activation molecule 7 (SLAM7) was examined in carotid plaque vs. intact tissue, in advanced plaque vs. early atherosclerotic tissue, and SLAMF7 protein expressed significantly higher in the unstable plaques than that in the stable plaques, especially in the CD68-positive macrophages. Depletion of SLAMF7 in plaque-derived macrophages induced a suppressed secretion of proinflammatory cytokines, and inhibited proliferation of vascular smooth muscle cells. These data provide emerging evidence that SLAMF7 could be a target of potential therapeutic intervention in carotid AS.

## Introduction

Atherosclerosis (AS), characterized by atheromatous plaques in the intima of the large arteries, is an underlying cause of many cardiovascular diseases, including heart attack and stroke, and the leading cause of death and morbidity among adults worldwide [1]. It is widely accepted that AS is a complex multifactorial disease with genetic, epigenetic and environmental factors associated to the disease progression [2]. In recent years, numerous genome-wide association studies with AS have identified several risk loci for AS associated genes [3]. While these studies provided an initial look into the genetic architecture of AS, most of the loci showed inconsistent results between different studies [3]. Genetic risk factors can explain only a little portion of the observed inheritance of AS [4]. The small genetic variance for AS could be due to environmental factors and/or epigenetic regulation. Indeed, emerging studies have implicated epigenetic regulation in the etiology of AS, including DNA methylation, histone post-translational modifications and microRNAs [5]. DNA methylation is the most stable epigenetic mark that confers persisting changes in gene expression [6,7]. Although a significant level of decreased genomic DNA methylation was identified in human atherosclerotic lesions [8], a further exploration of DNA methylation in regulating gene transcription in a cell type- and stage-specific manner is warranted, and such study will shed light on understanding the biological processes associated with AS severity and plaque stability.

In this study, we tempted to systematically identify key regulators in AS by integrating functional genomic, DNA methylation, and higher order pathological phenotypic data. We performed whole genome RNA sequencing and DNA methylation analysis of carotid plaques from late-stage AS patients and age-/gender-matched healthy individuals that were not exhibiting AS risk factors. The gene expression changes that follow as a result of differentially methylated regions in proximity to the gene promoter were identified. In comparing with public available AS cohorts, those genes shown a consistent differential expression pattern between plaque vs. distant intact tissue and advanced vs. early atherosclerotic artery tissue were further associated to either macrophages or smooth muscle cells. Protein expression of the genes were verified in clinical plaque samples which were classified as complex lesion or “unstable” plaque (UnS) and noncomplex or “stable” plaque (S), as UnS plaques have been reported to be more often associated with cardiovascular events [9]. Signaling lymphocytic activation molecule 7 (SLAM7), a member of SLAM family receptors, was identified to express significantly higher in the UnS plaques than in the S plaques, especially enriched in the CD68-positive macrophage area. Functional study revealed that SLAMF7 not only played a stimulatory role in releasing proinflammatory cytokines in AS macrophage, but also mediated the proliferation suppression on vascular smooth muscle cells (VSMCs), which contributes to the progression of unstable AS.

## RESULTS

### Patient demographic information

**Figure 1:**
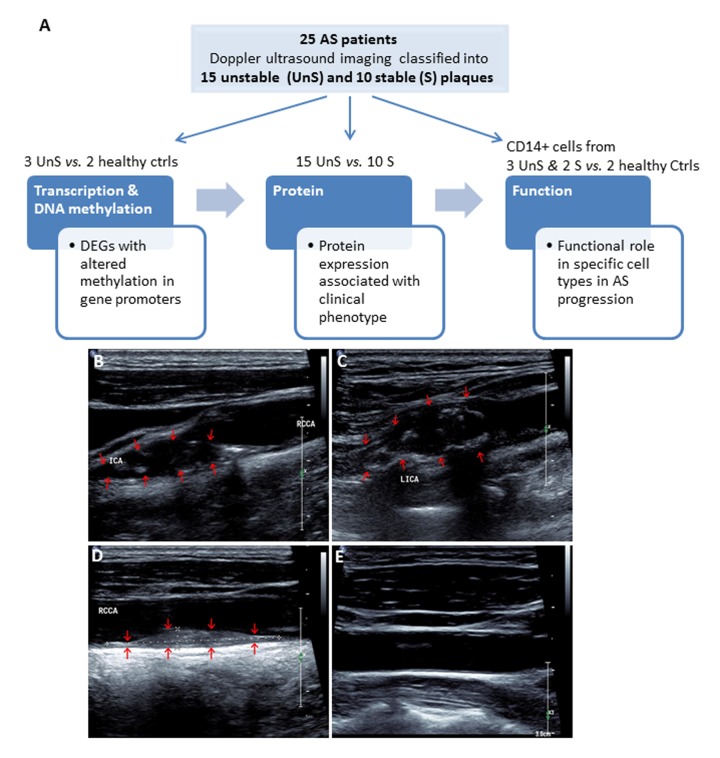
(**A**) Work flow of the present study. A total of 25 consecutive patients undergoing carotid endarterectomy for AS, and 2 healthy subjects were enrolled. All patients underwent Doppler ultrasound imaging for the carotid plaques before endarterectomy, and the carotid atherosclerotic plaques were classified as complex lesion or “unstable” (UnS) and noncomplex or “stable” (S) plaques. Two UnS plaque samples were used for RNA-seq (one patient and one age-/gender matched healthy control), 5 for DNA methylation (2 patients and 2 healthy controls, including the same samples for RNA-seq, and bilateral carotid plaques from one same patient), 25 for protein and histological analysis (15 UnS and 10 S plaques), and 5 fresh samples to isolate CD14+ myeloid cells (i.e. monocytes and macrophages) for functional studies, including siRNA transfection and cytokines analysis for macrophages, and proliferation and cell cycle analysis for vascular smooth muscle cells (VSMCs). (**B**-**E**) Representative Doppler ultrasound images showing UnS (**B**, **C**) and S (**D**) carotid artery plaques, and a normal carotid artery (**E**). Plaques are demonstrated by red arrows.

A total of 25 consecutive patients undergoing carotid endarterectomy for AS were enrolled in Liaocheng People's Hospital of Taishan Medical University between June 2010 and Jan 2016 (Fig. 1A). All patients underwent Doppler ultrasound imaging for the carotid plaques before endarterectomy. The carotid atherosclerotic plaques were classified as complex lesion or UnS plaques and noncomplex or S plaques (Fig. 1B-D). Image of a healthy subject carotid artery demonstrates intima-media with no plaques and normal blood flow (Fig. 1E).

All participants were human immunodeficiency virus- and hepatitis B virus-negative and did not exhibit inflammation or liver and kidney diseases. AS is often accompanied by lipid abnormalities; AS patients in the enrolled study demonstrated above-than-normal ranges of the following factors: total cholesterol (TC), total triglycerides (TG), high-density lipoprotein (HDL), apolipoprotein A and apolipoprotein B, and a lower than normal range of low-density lipoprotein (LDL). However, patients with UnS plaques or S plaques didn’t show significant difference of most factors except TG (Table 1), although they were matched according to gender, age, smoking habit and alcohol consumption. The healthy control subjects did not have abnormal lipid metabolism.

**Table 1:** Demographic characteristics of the study subjects.

Characterization	Groups		P value
	Patients with UnS plaque (N=15)	Patients with S plaque (N=10)	
Gender ratio (male/female)	13/2	8/2	1.000
Age (years)	64.47±7.41	66.8±7.39	0.448
Alcohol (Y/N)	7/8	2/8	0.229
Smoking (Y/N)	9/6	4/6	0.428
TG (mmol/L)	1.82±1.05	1.15±0.61	0.084
TC (mmol/L)	4.78±1.13	3.93±0.86	0.057
HDL (mmol/L)	1.16±0.22	1.06±0.25	0.308
LDL (mmol/L)	3.05±0.90	2.31±0.50	0.028
Apolipopeotein A (g/L)	1.12±0.17	1.12±0.16	0.991
Apolipopeotein B (g/L)	0.94±0.21	0.81±0.13	0.090

Abbreviation: TC: total cholesterol; TG: total triglycerides; HDL: high-density lipoprotein; LDL: low-density lipoprotein

### Identification of differentially expressed genes (DEGs) associated with differentially methylated CpG islands

**Figure 2:**
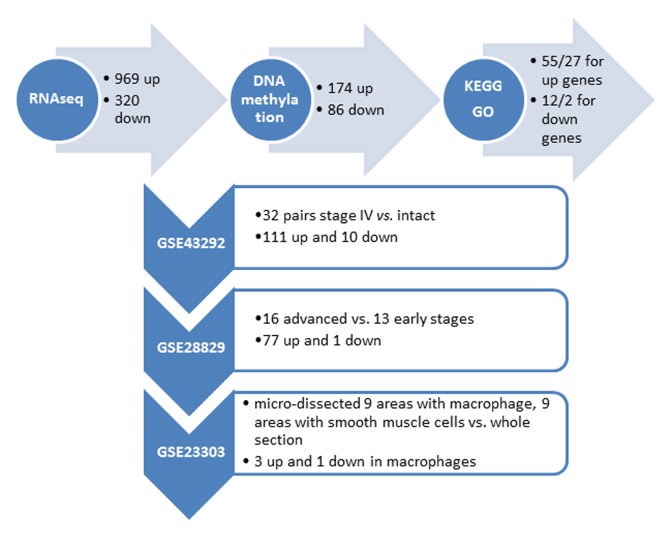
**Summary of the identified genes with altered DNA methylation in the promoter region** **from our own (top horizontal panel) and different independent datasets.** (Vertical panel, GEO numbers are listed at the left side, the brief description of the dataset and the identified genes are listed at the right side).

We performed RNA-seq profiling of 2 carotid artery samples (one patient and one age-/gender matched healthy control), and whole genome DNA-methylation profiling of 5 carotid artery samples (2 patients and 2 healthy controls, including the same samples for RNA-seq, and bilateral carotid plaques from one same patient) (Fig. 1A). Raw RNA-seq data were mapped to human reference genome hg19 downloaded from UCSC genome browser. The concordant pair alignment rate for each sample was 81.6% and 81.1%, respectively. Gene fragments per kilobase million (FPKM) values were calculated by Cufflinks (v2.2.1) using default parameters. DEGs were selected as |log2(fold change)| > 3. Overall 1289 DEGs were selected (969 up-regulated and 320 down-regulated) (Fig. 2).

Raw DNA methylation data were mapped to human reference genome hg19 using Bismark (v0.17.0). There were around 433M, 393M, 463M, 375M and 369M paired sequence reads from the 5 samples, respectively. Among them, around 227M, 193M, 249M, 260M and 247M have unique best hits on the reference genome. The mapping efficiencies (unique best hits/total sequence pair) were 52.31%, 49.05%, 53.84%, 69.28% and 66.86%. The overall methylation rates of CpG context were 82.06%, 80.99%, 80.14%, 79.11% and 78.87%. The methylation rates of CHG context were 1.50%, 1.60%, 1.73%, 1.14%, and 1.10%. The methylation rates for CHH context were 1.47%, 1.57%, 1.70%, 1.18% and 1.12%. Because the methylation rates of CHG and CHH were very low, we only investigated the methylation changes on CpGs of the promoter regions of the DEGs found by RNA-seq analysis. The promoter region was defined as 1500bp before and 500bp after the transcription start site (TSS) of each gene. Fisher test was used to identify statistic significant changes on the methylation ratios. Among the 1289 DEGs, 174 genes are up-regulated in AS which have hypo-methylated CpGs in the promoter regions and 86 genes are down-regulated in AS which have hyper-methylated CpGs in the promoter regions (Fig. 2) (Supplementary Tables 1 and 2).

### Kyoto Encyclopedia of Genes and Genomes (KEGG) pathway and gene ontology (GO) annotation of the DEGs

KEGG pathway and GO term enrichment analysis were conducted using DAVID Bioinformatics Resources 6.7 for both gene lists. Overall, 12 and 2 pathways as well as 55 and 27 GO terms with p<0.05 were identified for the up- and down-regulated genes, respectively (Supplementary Tables 3 and 4), dominated by immune response, inflammatory processes, and calcium signalings. These results are concordant to the current understanding that crosstalks between bone, vasculature, and the immune system play a crucial role in the propagation of AS or vascular calcification [10].

### Expression of the DEGs in other independent AS cohorts

We further utilized several independent cohorts with gene expression profiling of AS to explore clinical relevant biomarkers from our gene list. First, the gene expression dataset GSE43292 was analyzed. This gene expression study was conducted from pieces of carotid endarterectomy collected in 32 hypertensive patients. The samples contained media and neo-intima without adventitia. They were paired, including for each patient one sample of the atheroma plaque (stage IV and over of the Stary classification) containing core and shoulders of the plaque, and one sample of distant macroscopically intact tissue. Among the 174 up + 86 down genes in our list, the differential expression status of 111 up and 10 down genes were identified in atheroma plaque vs. intact tissue (p<0.05) (Fig. 2) (Supplementary Table 5).

Second, the gene expression dataset GSE28829 was analyzed. This gene expression study was conducted from patients’ atherosclerotic carotid artery segments, from 13 pathological early (intimal thickening and intimal xanthoma) and 16 advanced (thin or thick fibrous cap atheroma) lesions. Among the 111 up + 10 down genes that are differentially expressed between atheroma plaque vs. intact tissue, the differential expression status of 77 up and 1 down genes were identified when comparing advanced with early atherosclerotic artery tissue (p<0.05) (Fig. 2) (Supplementary Table 6).

Third, the gene expression dataset GSE23303 was analyzed. This gene expression was conducted from laser capture micro-dissected smooth muscle cells and macrophages from carotid plaque sections. The atherosclerotic arterial wall is implicated with activation of macrophages and proliferation of vascular smooth muscle cells. We examined that only 3 out of the 77 up and 1 down gene have a distinct expression pattern in macrophages in comparing with a pooled RNA from whole sections, i.e., SLAMF7 (p=0.02), TMEM176B (p=0.04) and OSBPL3 (p=0.04) are up-regulated in macrophage, and ATP1A2 is down-regulated in macrophage (p=0.03).

### Protein expression of SLAMF7 in clinical carotid atherosclerotic plaques

**Figure 3:**
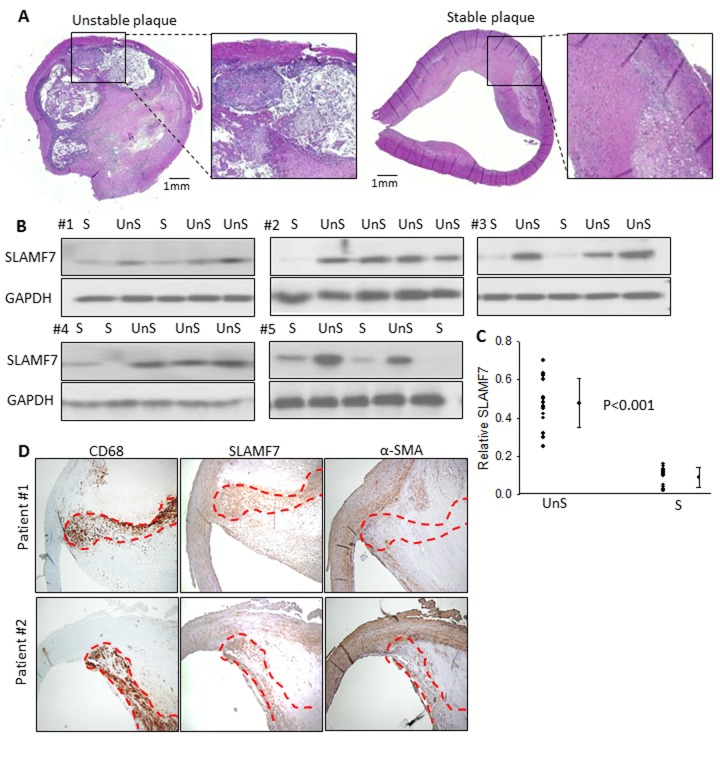
**SLAMF7 expresses significantly higher in the UnS plaques than in the S plaques.** (**A**) Representative histological H&E staining of UnS and S plaques in our study. (**B**) Protein expression of SLAMF7 was evaluated in the total of 15 UnS and 10 S plaques. Western Blot analyses were performed in 5 batches (#1-5) when patients were enrolled consecutively, GAPDH was used as internal control. (**C**) Quantification of the SLAMF7 expression in UnS vs. S plaques of the Western Blot. P<0.001, by Rank sum test. (**D**) Serial tissue sections from 2 individual patients were stained with anti-CD68, SLAMF7 and α-SMA antibodies. Red dash line segmented CD68+ macrophage area.

To further explore the clinical relevance of these genes, carotid atherosclerotic plaques, from 25 patients were used to examine the protein expression of SLAMF7. These plaques were classified as complex lesion or UnS plaques (n=15) and noncomplex or S plaques (n=10) (Fig. 1B-D and 3A). UnS plaques have been reported to be more often associated with cardiovascular event [9]. SLAMF7 was identified to express significantly higher in the UnS plaques than in the S plaques (p<0.001, by Rank sum test) (Fig. 3B-C), especially enriched in the CD68-positive macrophage area (Fig. 3D). SLAMF7 is a member of signaling lymphocytic activation molecule family receptors expressed on immune cells playing critical roles in immune regulation. Its role in AS has barely been investigated.

### SLAMF7 in atherosclerotic monocytes and macrophages

**Figure 4:**
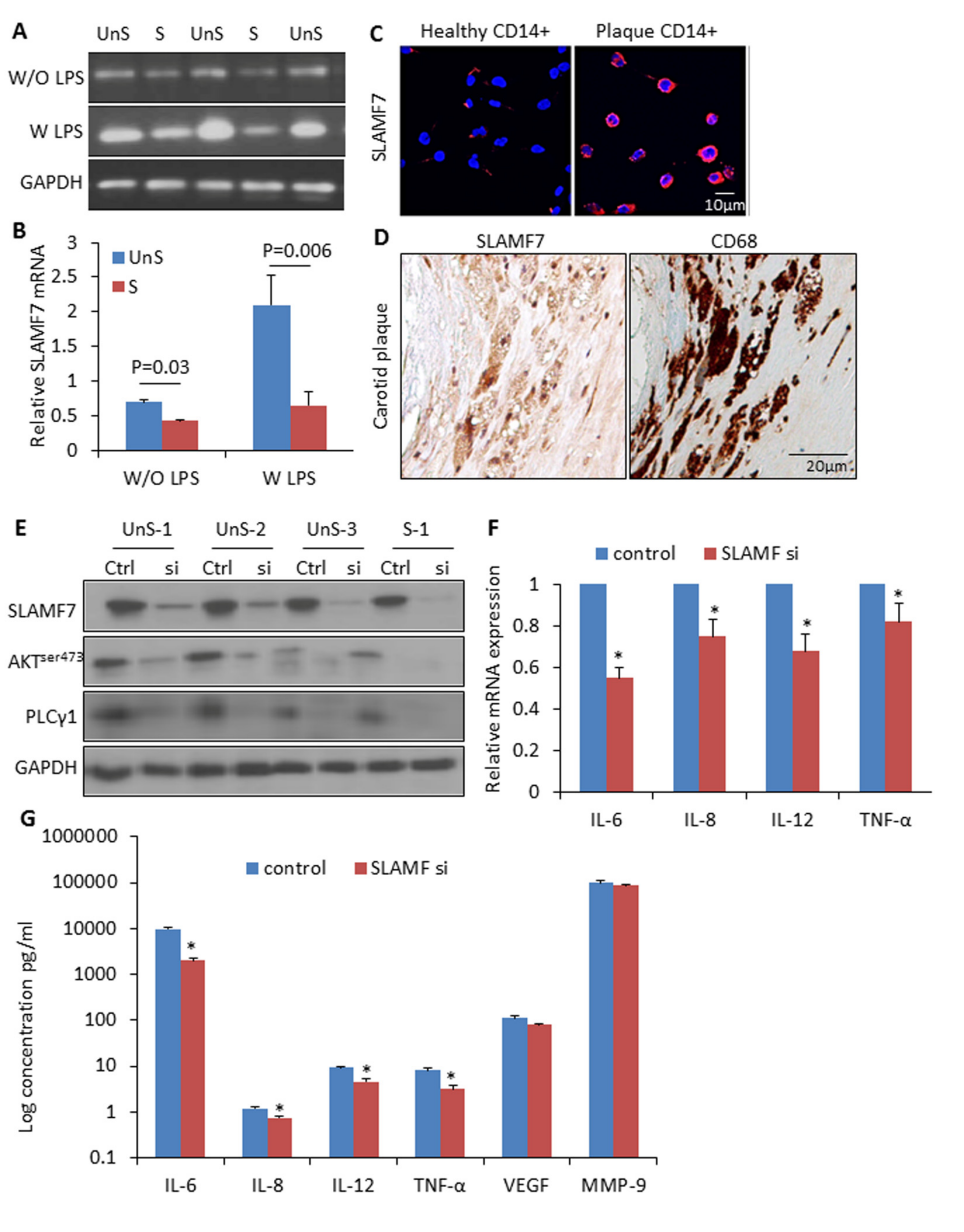
**High expression of SLAMF7 in CD14+ plaque monocytes stimulates proinflammatory cytokine production.** (**A**) CD14+ macrophages were isolated from human atherosclerotic plaques, and cultured for 24 hours. SLAMF7 mRNA was detected by RT-PCR with or without LPS (10ng/ml) stimulation for 3 hours. (**B**) Quantification of the SLAMF7 mRNA expression in UnS vs. S plaques. P=0.03 W/O LPS, P=0.006 W LPS, by student’s t test. (**C**) Immunofluorescent staining of SLAMF7 in CD14+ cells from healthy donor and one UnS plaque. (**D**) Immunohistochemistry staining of SLAMF7 in adjacent sections with anti-SLAMF7 and CD68 antibodies, respectively. (**E**) Transduction with specific siRNAs targeting SLAMF7 (SLAMF7 siRNA, 200nM) in the plaque-derived CD14+ cells for 24 hours resulted in a significant suppression of protein expressions of SLAMF7, AKT^ser473^ and PLCγ1 by Western Blot analyses. (**F-G**) Transduction with SLAMF7 siRNAs in the plaque-derived CD14+ cells for 24 hours resulted in a significant reduction of proinflammatory cytokines IL-6, IL-8, IL-12 and TNF-α measured by RT-PCR (F) and ELISA (G). Independent experiments were done on CD14+ monocytes from 4 different donors and the results are shown as mean ± SD. *P<0.05, vs. corresponding control, by student’s t test.

We isolated CD14+ myeloid cells (i.e. monocytes and macrophages) from human atherosclerotic plaques and healthy donors’ blood, and cultured them with or without lipopolysaccharides (LPS) (10ng/ml). SLAMF7mRNA showed a 1.6-fold higher expression in the plaque CD14+ myeloid cells than in the control cells without LPC stimulation, and a 3.2-folde higher after LPS induction for 3 hours (Fig. 4A-B). SLAMF7 protein was examined on both cellular surface and cytoplasm of the plaque CD14+ cells in vitro and in vivo (Fig. 4C-D). Transduction with specific siRNAs targeting SLAMF7 in the UnS plaque-derived CD14+ cells for 24 hours resulted in a significant reduction of proinflammatory cytokines interleukin-6 (IL-6), IL-8, IL-12 and tumor necrosis factor alpha (TNF-α) (p<0.05), mediated by the SLAMF7 downstream protein kinase B (PKB, also known as AKT) and phosphoinositide phospholipase C (PLCγ1) signalings (Fig. 4E-G).

### SLAMF7 mediated VSMC proliferation and apoptosis

**Figure 5:**
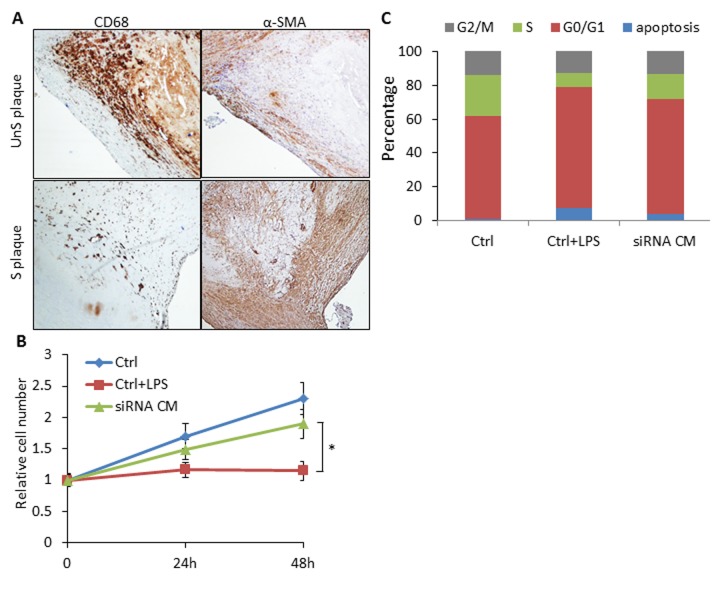
**SLAMF7 mediates reduced proliferation of VSMCs in the UnS plaque.** (**A**) Representative immunohistochemistry staining images showing lower prevalence of VSMCs compared to the CD68+ macrophages in UnS plaques than in S plaques. (**B**) 5 × 10^4^ VSMCs were plated in 6-well plates, serum starved overnight, and cultured with conditioned medium (CM) from human CD14^+^ macrophages. Ctrl: VSMCs were treated with CM from CD14+ cells transfected with control siRNA; Ctrl+LPS: VSMCs were treated with CM from LPS stimulated control siRNA CD14+ cells; siRNA CM: VSMCs were treated with CM from SLAMF7 siRNA CD14+ cells stimulated with LPS. After 24 and 48 h, cells were harvested and counted by hemocytometer. *P < 0.05 vs. Ctrl+LPS. Each data point in all graphs represents the mean±SD of 3 independent experiments. (**C**) Cell cycle distribution of VSMCs treated with CM.

UnS plaques were characterized by lower prevalence of VSMCs compared to the CD68-positive macrophages in contrast to what observed in S plaques [9], especially in the shoulder area of the plaque where rupture occurs most commonly [11] (Fig. 5A).To explore whether SLAMF7 from macrophages may interfere with VSMCs biology, we collected conditioned medium (CM) from SLAMF7siRNA transduced CD14+ myeloid cells from 3 UnS plaques (siRNA CM), and assessed the effects of CM on VSMCs proliferation and cell cycle. We found that CM from the CD14+ myeloid cells activated with LPS induced the inhibition of VSMC proliferation, as tested by cell count. Conversely, VSMCs treated with SLAMF7siRNA CM showed a significant increase of cell proliferation at both 24 and 48h compared to LPS control medium (Ctrl+LPS) (p<0.05) (Fig. 5B). Cell cycle analysis further indicated that SLAMF7siRNA CM induced the increase of cell percentages in S-phage (from 8.37±1.82% to 15.05±2.36) and decrease in both G0/G1 and apoptotic phases (from 71.67±2.62% to 67.91±2.94% for G0/G1 and from 7.38±2.45% to 3.72±1.28% for apoptosis) (Fig. 5C). These results indicate that SLAMF7 plays a stimulatory role in releasing proinflammatory cytokines in plaque macrophages, and further affects the proliferation and apoptosis of VSMCs, contributing to the progression of unstable AS.

## DISCUSSION

By using a systematic approach in integrating genome-wide DNA methylation, gene expression, pathological phenotypic data, and functional study on primary-cultured cells, we identified SLAMF7 as a key regulator of carotid AS disease progression. In specific, the up-regulation of SLAMF7 gene expression in carotid plaque followed as a result of hypo-methylation in the SLAMF7 gene promoter. The involvement of epigenetic mechanisms in the development of AS has been extensively investigated [12]. However, our study is the first time to demonstrate that the methylation pattern of SLAMF7 is involved in carotid AS by doing functional experiments. The methylation of SLAMF7 gene has been linked to inflammation-related genes under chronic stress [13], which potentially mediates the release of several proinflammatory cytokines from the CD14+ myeloid cells from patients’ atherosclerotic plaques, and inhibit VSMCs cell proliferation. These data provide emerging evidence that SLAMF7 could be a target of potential therapeutic intervention in carotid AS.

SLAMF7 was originally identified as a natural killer (NK) cell-associated surface molecule [14]. Subsequently, it was shown to be expressed on lymphocytes and monocytes [15]. More recently, SLAMF7 was identified as a novel M1-like cell surface marker of human macrophage [16]. In context of respective immune response, macrophages are polarized to specific functional properties, often referred to as M1-like and M2-like phenotype [17,18]. The homeostasis between pro-inflammatory M1 and anti-inflammatory M2 macrophages has been deemed crucial in athrogenic initiation, progression and symptomatology [19]. In our study, we identified a consistent epigenetic DNA methylation-driven elevated expression of SLAMF7 in carotid plaque vs. intact endarterium; in advanced vs. early atherosclerotic plaque; in unstable plaques vs. stable plaques. Macrophages as well as neutrophils and lymphocytes have been described in the plaques [20], but macrophages remain the most abundant cell type, and our cultured CD14+ cells contain mostly macrophages [21]. Epigenetic mechanism has been reported to stimulate M1 polarization in AS [22]. Plaque development may originate not only from persistent inflammation (M1 macrophage response), but also from inadequate anti-inflammatory responses (M2 macrophage response). If the pattern of SLAMF7 expression could represent the kinetics of M1 macrophage in lesion formation and disease progression, our results thus more favor the detrimental role of M1 macrophage in AS. Furthermore, M1 macrophages have been demonstrated to dominate the rupture-prone shoulder regions of plaques [19], and SLAMF7 was examined to enrich at the same location, indicating the association of SLAMF7 with AS severity and plaque stability. Nevertheless, the dynamics of SLAMF7 was not investigated from the same individual longitudinally, thus warrants further verification using animal models with AS.

VSMC is another important component in AS plaque, and a decreased number of VSMCs favors plaque rupture and thrombus formation [11]. SLAMF7 is a self-ligand, like most of the SLAM family members, exhibiting homophilic interactions, i.e., it recognizes as ligand of another SLAMF7 molecule on another cell [23]. However, we couldn’t find any direct evidence about the expression of SLAMF7 on VSMCs (data not shown). On the contrary, we demonstrated that SLAMF7 depletion could render the inhibited cell proliferation of VSMCs affected by the soluble factors released from the unstable plaque-derived CD14+ macrophages. The evidence that the conditioned medium from activated macrophages inhibit VSMCs proliferation supports the central role of macrophage-derived cytokines [24]. Our data depicted the role of SLAMF7 in a pathophysiological scenario of AS, in which the abundance of SLAMF7 in infiltrating CD14+ cells leads to the humoral inhibition of VSMCs proliferation and plaque instability.

The humanized anti-SLAMF7 monoclonal antibody elotuzumab [25,26] has been developed and shown promising efficacy in phase 1 and 2 trials on patients with refractory and relapsed multiple myeloma [27]. Even more interestingly, SLAMF7 has been recently revealed to be critical in phagocytosis mediating macrophages engulf and destroy haematopoietic tumor cells [28]. Phagocytosis has been well recognized as a crucial process involved in atherogenesis that may significantly affect the stability of the atherosclerotic plaque [29]. The potential effects of anti-SLAMF7 monoclonal antibody in AS, and the mechanism of SLAMF7-mediated phagocytosis in AS may worth future exploration.

The limitation of the current study was the small sample size of RNAseq and DNA methylation study. However, we tried to overcome this by adding the verification process using a series of public available cohorts at different stages of AS to sort out the most consistently changed genes, and further validated the protein expression on a reasonable collection of patient tissue sections.

## MATERIALS AND METHODS

### Ethics statement

The present study was reviewed and approved by the Ethics Committee of Liaocheng People's Hospital of Taishan Medical University. Written informed consent was obtained from the participants before the collection of any samples and the specimens were irreversibly de-identified. This research was performed in agreement with the principles outlined in the Declaration of Helsinki.

### Inclusion and exclusion criteria

Patients with AS were diagnosed by Doppler ultrasound examination of the carotid artery. The patients selected for surgery had either a high-grade stenosis (>70%) or an ulcerated lesion of a medium grade based on echo-Doppler analysis. The exclusion criteria were as follows: Individuals with moderate or severe valvular heart disease, previous or current cancer history, chronic liver disease, chronic renal failure or diabetes; and obese individuals. The healthy control group comprised of age- and gender-matched healthy individuals that were not exhibiting AS risk factors.

### Human specimens and study workflow

This study included a total of 25 consecutive patients undergoing carotid endarterectomy for AS in the Surgery Unit of Liaocheng People's Hospital, and 2 healthy subjects that died in car accidents and the carotid artery specimens were collected within 3 hours post death. Two samples were used for RNA-seq (one patient and one age-/gender matched healthy control), 5 for DNA methylation (2 patients and 2 healthy controls, including the same samples for RNA-seq, and bilateral carotid plaques from one same patient), 25 for protein and histological analysis (15 “unstable” and 10 “stable” plaques, see below), and 5 fresh samples to isolate CD14+ myeloid cells (i.e. monocytes and macrophages)(study work flow see Fig. 1A). Cross-sectional segments (5 mm) were taken along the course of each plaque. Segments were immediately snap-frozen.

### Plaque classification

Carotid atherosclerosis was assessed for all patients by carotid ultrasonography before endarterectomy. UnS plaques showed one or more of the following features: ulceration, irregular surface, presence of mobile thrombi on the plaque surface or intraluminal thrombus, predominant echolucency (hypoechoic plaques), and heterogeneity with substantial intraplaque-echolucent areas. S plaques were required to have all of the following features: smooth and regular surface and homogeneity and uniform echogeneity or dominant echogeneity with small areas of echolucency. All ultrasonographic examinations were performed by experienced ultrasonographers blinded to clinical and angiographic findings.

### RNA isolation, RNA-Seq library preparation and sequencing

As we described previously [30], total RNA was purified from the segmented plaques using the Qiagen RNeasy kit. RNA libraries were prepared with the Illumina TruSeq library kit as described by the manufacturer. Paired-end sequencing with 150-bp read lengths was performed on Illumina HiSeq X10 instrument. Raw RNA-seq data were mapped to human reference genome hg19 downloaded from UCSC genome browser. Gene FPKM values were calculated by Cufflinks (v2.2.1) using default parameters. Genes labeled no test were removed from following analysis. The differential expression results were based upon the fold-changes of the expression levels with statistical p-value <0.05. DEGs were selected as |log2 (fold change)| > 3. The result of the DE genes was clustered by the COG analysis using Fisher test and Chi-square test.

### Whole-genome DNA methylation sequencing and analyses

Genomic DNA was isolated from 5mg of plaque tissue, using the MasterPure™ DNA Purification Kit (Epicentre) according to the manufacturer’s instructions. Bisulfite treatment of genomic DNA was then performed using the Zymo EZ DNA Methylation Lightning Kit. The bisulfite-treated DNA was purified on a spin column and used to prepare the sequencing library using the EpiGnome™ Kit (Epicentre). Paired-end sequencing with 150-bp read lengths was performed on Illumina HiSeq X10 instrument. Methylation analysis was performed using Bismark (v0.17.0). In summary, FASTQ files were quality-filtered and adapter sequences trimmed using Trimmomatic. A bisulfite-converted UCSC hg19 reference genome file was generated using Bowtie, and the EpiGnome library sequence data were aligned to the reference genome. Methylation information was extracted for those DGEs identified from the RNA-seq analyses. The promoter region was defined as 1500bp before and 500bp after the TSS of each gene. Fisher test was used to identify statistic significant changes on the methylation ratios.

### Immunohistochemical staining

Plaques obtained from human carotid endarterectomy were fixed in formalin, embedded in paraffin and sectioned at 5μm with a rotary microtome. Sections were incubated with anti-CD68, α-actin and SLAMF7 primary antibodies (Abcam Biotechnology company, USA) at room temperature and subsequently exposed to specific secondary antibodies (Sigma-Aldrich, USA). Whole-slide montage images were taken by the automatic microscope ImageExpress (Molecular Device, USA). Images were digitalized and the relative staining areas were quantified by Image J. A histology score (H score) was calculated by multiplying the fraction of positively stained cells (percentage) by staining intensity (0, 1+, 2+, or 3+) [31]. Intensity of immunoreactivity was scored (0 and 1+ indicates negative; 2+, indeterminate; and 3+, positive for overexpression), and the percentage of staining positive was visually estimated by certified pathologist at the Pathology Department of Liaocheng Hospital.

### Cell culture, conditioned media and siRNA transfection

CD14+ myeloid cells (i.e. monocytes and macrophages) were isolated from human atherosclerotic plaques using magnetic beads bound to anti-CD14 antibody (Invitrogen, USA), as previously described [32]. Alternatively, peripheral blood monocytes (PBMCs) were separated by density gradient centrifugation of healthy donors. These cells were cultured overnight in fully supplemented DMEM in a 37°C 5% CO_2_ incubator. siRNAs targeting SLAMF7 (Invitrogen, USA) and HiPerFect transfection reagent (Qiagen, USA) were used to knockdown the protein expression of SLAMF7 in the cultured CD14+ cells. Conditioned media was collected from cultured cells, stimulated with 10 ng/mL of LPS for 24h [33]. Human aortic smooth muscle cell line was purchased from LifeLine Cell Technology (Shanghai, China).

### Cytokines quantification

CD14+ myeloid cell conditioned media (with SLAMF7siRNA or control siRNA transfection) were screened for the concentration of IL-1β, IL-6, IL-10, IL-12 (p70), TNF-α, vascular endothelial growth factor (VEGF), matrix metalloproteinase 9 (MMP-9). Measurements were performed with the Bio-Plex human cytokine kit from BioRad (BioRad, USA), according to the manufacturer's protocol.

### Western blot analysis

Human atherosclerotic plaques or siRNA-transfected CD14+ cells were lysed in ice-cold RIPA-SDS buffer. Protein concentration was determined using BCA assay kit (Boster Technology, China). Equal amounts of total protein extracts were electrophoresed on 4–12% SDS-PAGE gel and transferred to PVDF membrane (Millipore Corporation). The membranes were blocked in Tris buffered saline containing 0.002 g/L Tween 20 (TBST) and 0.05 g/L nonfat dry milk. After blocking, the membranes were washed three times in TBST and then incubated overnight at 4°C in TBST containing 5% BSA with primary specific antibody: anti-SLAMF7 (ab67135, Abcam biotech), anti-PLCγ 1(sc7290, Sant Cruz Biotechnology), and anti-pAKT (Ser^473^)(#9271s, Cell Signaling Technology). The blots were washed three times in TBST, incubated in appropriate HRP-conjugated secondary antibodies (1:2000, Santa Cruz Biotechnology, Inc.,), dissolved in TBST containing 5% nonfat dry milk and incubated for 1 h at room temperature. After 3 additional washes with TBST, immuno-reactive bands were visualized by enhanced chemi-luminescence using the ECL-plus detection kit (Boster Technology, China) and quantified by using ImageJ software.

### Cell proliferation and cell cycle analysis

Serum starved VSMCs (50,000 cells/well) were treated with cell-free supernatants of cultured CD14+ myeloid cells (with SLAMF7siRNA or control siRNA transfection). After 24 and 48 h, cells were detached from the plates, and counted on hemocytometer. Distribution of cell cycle population was analyzed by flow cytometry according to the following standard procedure. In brief, cells were harvested using trypsin, washed with PBS, and gently resuspended in 250 μL of hypotonic fluorochrome solution (PBS, 50 μg propidium iodide, 0.1% sodium citrate, and 0.1% Triton X-100) with RNase A (100 units/mL). The function of the fluorochrome solution is to stain cell nuclei. The DNA content was analyzed on the FACS Calibur Flow Cytometer (Becton Dickinson). Twenty-thousand events were analyzed per sample and the cell cycle distribution was determined based on DNA. All analyses were repeated in triplicate.

### Statistical analysis

Patient demographic data were expressed as mean+SD for continuous variables, and as frequency for categorical variables. Any significant differences between the experimental and control groups were assessed using t-tests, chi-square tests and fisher exact tests, as appropriate. The t-tests and fisher exact tests were also used to analyze the IHC and cytokine quantification data, respectively. P<0.05 was considered to be statistically significant. The commercial software SigmaPlot 11.0 (Systat Software, Inc., San Jose, CA) was used for all statistical analysis.

## Supplementary Material

Supplementary Tables
