# Supplementary material for: Integrated DNA methylation and gene expression analysis identifies SLAMF7 as a key regulator of atherosclerosis
Source: Aging (Albany NY). 2018 Jun 13;10(6):1324–37. doi: 10.18632/aging.101470 (PMC6046250; doi:10.18632/aging.101470)
Supplement: Supplementary Tables [file aging-10-101470-s001.pdf]

SUPPLEMENTARY MATERIAL

Supplementary Table 1. Genes up-regulated in AS with hypo-methylated CpGs in the promoter regions

Gene_Symbol	mRNA_log2Fold (AS/control)	%Methy_Diff (AS-control)	methy_fisher_p	methy_FDR
SPP1	12.1995	-14.09437219	0.00114708	0.00598555
TRAC	10.8889	-15.14144606	3.76E-06	3.96E-05
APOC1	10.6362	-0.614550652	4.13E-05	0.00033242
CCL3	8.49961	-7.24090593	0.02986314	0.08984943
LINC00900	8.38495	-5.571183584	0.02537851	0.07931603
TXLNG2P	8.19567	-15.74460724	1.39E-05	0.00012962
HES2	8.09795	-18.50904778	0.0040868	0.01730754
BC040631	7.92439	-10.83517548	4.60E-05	0.00036382
TLR7	7.85045	-17.7032105	0.00097932	0.0052411
CXCR3	7.78663	-25.44959802	0.01752354	0.05871114
GZMA	7.53397	-37.65147584	5.86E-07	7.42E-06
EOMES	7.40862	-10.67027489	1.14E-07	1.67E-06
KMO	7.08425	-2.96941286	0.01129243	0.04095411
SLAMF7	7.0626	-18.42986699	5.26E-05	0.00041041
LOC100506776	6.90242	-9.115560366	0.02979639	0.08976024
CD52	6.79467	-34.45675627	5.11E-08	8.52E-07
LILRB4	6.74963	-20.43933227	0.00921425	0.03461066
LY9	6.67798	-22.63815167	7.26E-07	8.96E-06
TDO2	6.65902	-4.605610294	0.02270635	0.07292916
STAP1	6.52246	-15.82994896	2.57E-05	0.0002195
FCGR3A	6.50852	-9.241461931	0.00451974	0.01888298
IGSF6	6.40885	-12.31232729	0.00374737	0.01612971
SAMD3	6.36852	-11.93853896	0.0001327	0.00094693
DCSTAMP	6.19836	-21.41787974	2.36E-07	3.20E-06
KCNA3	6.1858	-20.115835	3.59E-08	6.12E-07
TMEM26	6.09135	-12.93714587	0.00041833	0.00248634
ITGAL	6.03052	-13.07823613	6.84E-06	6.86E-05
KLRC1	5.94987	-3.990151803	4.18E-05	0.00033402
DQ658414	5.84523	-3.373964093	0.00161661	0.00798076
LYZ	5.84024	-19.4847124	5.15E-06	5.30E-05
ARL11	5.77745	-8.245751718	0.00013698	0.00097172

RAB42	5.7742	-7.163643235	0.01843926	0.06090477
TRAT1	5.76121	-8.466366097	7.66E-06	7.53E-05
P2RX7	5.75476	-16.26746957	2.01E-08	3.56E-07
MYBL2	5.69482	-28.11865912	0.00010533	0.00076975
CD8A	5.68958	-13.12367894	0.00183734	0.00903356
FCGR1C	5.67327	-23.10791611	0.00024918	0.0016031
CD96	5.53367	-12.94060914	9.09E-12	2.75E-10
CYSLTR2	5.39354	-19.86475214	4.68E-05	0.00036893
CD4	5.38596	-16.43867651	0.00024487	0.00158806
EMR4P	5.37993	-20.02069884	0.01676856	0.05665243
OSCAR	5.31551	-0.932093835	0.00723399	0.02786467
CD2	5.26337	-20.68318378	3.71E-07	4.79E-06
SLAMF8	5.24399	-27.77007314	0.01768823	0.05918094
PDE6B	5.19618	-22.03391053	5.91E-08	9.53E-07
KLRK1	5.18093	-11.8750407	0.00016549	0.00114052
CCL5	5.16838	-23.89408369	0.00028373	0.00180141
C15orf48	5.14186	-19.0811632	1.24E-11	3.67E-10
CD48	5.10651	-12.78881535	0.01075954	0.03925691
SUCNR1	5.0365	-10.69821356	0.00048397	0.00284158
MS4A14	5.0305	-8.9192849	0.01045855	0.03833217
MT1H	5.01515	-30.43015677	0.00024912	0.0016031
PIK3CG	4.98865	-31.92217984	0.00034729	0.00211608
S1PR5	4.98161	-2.12528141	0.00446743	0.01869671
LILRB1	4.91497	-15.45219996	0.00322032	0.01431978
IL2RG	4.88028	-20.33311777	7.59E-09	1.43E-07
PARVG	4.87332	-40.22913848	3.91E-19	3.26E-17
ADAM28	4.85682	-19.77653358	1.90E-08	3.40E-07
AK055623	4.84076	-47.13293651	0.00084423	0.00462035
SCUBE1	4.82531	-9.759671545	0.01021424	0.03760767
AK307192	4.81634	-32.57472527	0.02321301	0.07398192
LAIR1	4.76632	-5.551449059	0.03273751	0.0962236
TCRVB	4.75972	-10.43300887	0.00351669	0.01535539
TRAF3IP3	4.7484	-27.14939455	2.61E-06	2.95E-05
ARL4C	4.72364	-23.84440419	1.08E-19	9.30E-18
SRGAP3	4.70534	-6.262804585	0.00519573	0.02133868

EVI2A	4.68699	-36.37913582	3.51E-29	9.42E-27
SASH3	4.68156	-16.25641923	0.00944487	0.03531244
CD33	4.55821	-3.029596065	0.03120322	0.09261422
GAL3ST4	4.55167	-33.95360922	3.49E-05	0.00028494
LILRA1	4.52312	-13.8993262	0.02217335	0.07151644
DOK2	4.50332	-30.81529582	0.00059409	0.00342986
C1QA	4.49405	-30.67581373	0.00219422	0.01048445
LRRC33	4.43969	-43.72235736	3.16E-08	5.46E-07
VAMP8	4.43364	-18.39795918	0.00616426	0.02464685
FABP5	4.42594	-18.96814532	1.76E-14	8.51E-13
ARHGAP8	4.39278	-10.97142587	8.33E-06	8.13E-05
TYROBP	4.37663	-17.42548029	0.03426166	0.09987229
RASL10A	4.37439	-3.820032334	4.80E-06	4.98E-05
DSC2	4.33136	-7.334668188	0.00291025	0.01318333
IKZF3	4.32523	-11.01793622	0.02473971	0.07782232
BC044305	4.3238	-13.3000148	0.02166587	0.0701603
BUB1B	4.3044	-7.485262964	0.0081223	0.0309415
ASF1B	4.28317	-26.54715856	0.00733901	0.02817947
MS4A7	4.25979	-17.74404332	0.02164803	0.0701603
NR1H3	4.22868	-33.74885392	0.00696498	0.02695725
BC016366	4.21897	-7.551957022	0.01537879	0.05306888
CCRL2	4.18439	-21.34824714	5.84E-09	1.15E-07
RCAN3	4.18389	-9.482672267	0.00669409	0.02611775
TAGAP	4.18111	-33.66286435	2.20E-35	1.06E-32
KRT8	4.15309	-4.868873686	0.00365063	0.01582594
TRGV3	4.06026	-6.10792293	0.00601718	0.02413856
MKI67	4.03232	-20.34106639	1.63E-07	2.36E-06
DAPP1	4.02498	-30.71879509	2.79E-06	3.14E-05
PRKCQ	4.01839	-0.777225228	0.00932102	0.03495744
TNFAIP8L2	3.9839	-31.60703406	2.66E-08	4.63E-07
FTCDNL1	3.9612	-15.86552059	8.36E-08	1.30E-06
C1orf233	3.95945	-14.05194805	0.03392181	0.09922232
OSBPL3	3.93382	-17.376593	3.97E-11	1.07E-09
LGALS2	3.93287	-1.765053829	0.0162522	0.05529404
CCR2	3.9244	-13.53552769	0.00557792	0.02263925

LILRA2	3.905	-48.17469295	0.0004399	0.00259542
GPR65	3.84498	-30.58007789	3.08E-13	1.14E-11
PTPRC	3.84223	-31.74937863	5.29E-18	3.76E-16
SP140	3.82056	-3.278828167	0.00112724	0.00593056
AK310146	3.8189	-3.176734137	0.00063493	0.00362237
MS4A6A	3.80729	-5.053437334	0.02793566	0.08553969
KIF20A	3.79211	-2.129894788	0.01818105	0.06032917
TROAP	3.75663	-33.38726805	2.63E-11	7.40E-10
CXCR4	3.75612	-20.03639803	9.72E-16	5.11E-14
CD101	3.75262	-25.66616519	1.06E-07	1.58E-06
GPR34	3.75217	-13.92790518	0.00266617	0.01217836
TBXAS1	3.74512	-17.33333333	6.94E-06	6.91E-05
MLPH	3.74136	-9.881467007	0.00013495	0.00096011
TNFSF12-TNFSF13	3.7281	-10.69760101	0.03057096	0.09163713
BC033739	3.71782	-9.009674408	0.00295374	0.01333041
APBB1IP	3.70332	-12.64009518	4.27E-05	0.00033959
SLC22A18	3.68421	-23.28010834	1.37E-05	0.00012808
ABCC3	3.68024	-8.751725328	0.01406188	0.04922675
ACAP1	3.67648	-10.09145881	0.00352924	0.01538241
NCKAP5	3.66221	-6.778036546	0.01585874	0.0544146
VSIG4	3.60784	-52.51984127	0.00134267	0.00683771
APOBR	3.59592	-42.05801342	2.11E-06	2.43E-05
LPXN	3.53758	-7.567171264	7.09E-09	1.36E-07
KLRG1	3.52646	-21.20117465	1.26E-11	3.69E-10
PCED1B-AS1	3.49794	-31.24555733	1.85E-07	2.60E-06
ABRACL	3.48663	-3.512245549	0.00618386	0.02468443
LGMN	3.44317	-6.458715391	0.02761735	0.08488737
GDPD1	3.43712	-31.16917017	3.17E-05	0.00026182
TK1	3.43512	-28.95497127	0.00113708	0.00595366
NELL2	3.41114	-11.97206889	5.04E-06	5.21E-05
KYNU	3.39642	-20.08782959	1.48E-08	2.72E-07
GAS2L3	3.3908	-0.832057855	5.52E-08	8.96E-07
AX747844	3.38471	-17.75308223	1.14E-05	0.00010798
WAS	3.37141	-24.30304814	1.82E-10	4.59E-09
LAT2	3.37111	-14.33576121	3.38E-06	3.65E-05

CYTH4	3.36817	-41.69711506	2.36E-11	6.70E-10
LRMP	3.36346	-33.38239116	1.51E-12	5.01E-11
GPNMB	3.34435	-8.197992236	0.00442435	0.01855163
SYTL1	3.32729	-27.66281866	0.00020454	0.00135932
FGD6	3.32282	-14.67300487	9.76E-05	0.00072007
INPP5D	3.27805	-48.10515873	1.38E-14	6.79E-13
F2R	3.22673	-10.13072047	0.02975049	0.0897337
RGS1	3.22422	-16.05274249	0.00264987	0.01217836
CLDN23	3.21783	-17.45061286	0.03432913	0.09993041
DAPK1	3.2053	-9.680115714	0.01165589	0.0421459
MARCO	3.20137	-17.02638037	0.00047273	0.00278231
APOBEC3C	3.19451	-7.660605954	2.54E-06	2.88E-05
RUNX3	3.18907	-10.34632035	0.01878872	0.06183663
RGS18	3.15948	-21.74056408	3.19E-06	3.47E-05
GMFG	3.15697	-11.203363	1.85E-05	0.00016427
TMEM176B	3.14626	-10.21067502	8.55E-07	1.04E-05
GAPT	3.12984	-25.41499412	4.22E-19	3.41E-17
COL21A1	3.10813	-8.635582428	0.01564148	0.05382182
TLR5	3.10278	-14.27210278	0.01866033	0.06149774
RBM47	3.0979	-2.850369328	0.00030391	0.00191947
ABCA1	3.08113	-21.88259583	0.00018348	0.00123974
MS4A4A	3.07338	-19.77727354	9.28E-06	8.94E-05
RASSF7	3.07004	-36.8968254	2.25E-07	3.08E-06
AK095210	3.0609	-19.31335326	2.68E-13	1.01E-11
MELK	3.05437	-23.36211692	9.22E-09	1.73E-07
BX648270	3.02876	-8.03784996	0.00022626	0.0014873
BIRC5	3.0133	-2.178546549	3.71E-06	3.91E-05
KLRC4	up	-17.73070801	2.29E-13	8.78E-12
APOC1P1	up	-31.58963585	9.79E-08	1.49E-06
E02193	up	-9.426587093	1.56E-05	0.00014277
TRA	up	-23.88952064	0.00018987	0.00127939
BC055085	up	-9.276907176	0.00032708	0.00201839
TCRBV7S1A1N2T	up	-39.96869489	0.00201611	0.00975394
ATP6V0D2	up	-8.273059983	0.00367934	0.01592187
TIFAB	up	-1.754936584	0.01804886	0.06008188

AF086258	up	-2.131004724	0.02321121	0.07398192
TCRBV22S1A2N1T	up	-4.490161111	0.02489996	0.07812321
ZEB2_AS1_1	up	-1.732804233	0.03153068	0.09347145

Note: The ones with no fold change is because they do not express at healthy control sample (FPKM=0) but expressed at AS sample. So the log2(fold) is then calculated by log2(0) which is negative inf. The software outputs no value of fold change for these genes but actually they have significant fold change between AS and control.

Supplementary Table 2. Genes down-regulated in AS with hyper-methylated CpGs in the promoter regions.

Gene_Symbol	mRNA_log2Fold (AS/control)	%Methy_Diff (AS-control)	methy_fisher_p	methy_FDR
BC073897	-11.5986	6.121004238	0.007613895	0.02918861
AF086165	-10.3295	2.172995359	0.003874275	0.01649978
CNTFR	-9.29815	44.375	0.002201777	0.01048445
CSF3	-8.90856	21.02022977	0.001483699	0.00747723
AVPR1A	-8.8681	33.65998151	2.28E-27	5.01E-25
CXCL14	-7.92924	17.20634557	6.92E-07	8.59E-06
C6	-7.64111	7.906780256	0.00311002	0.01390598
AK125532	-7.38541	55.69677681	1.67E-57	1.35E-54
APOD	-6.85829	5.557003104	0.01648122	0.05591595
FAM180B	-6.59223	16.67726967	0.000140959	0.00099411
MT1M	-6.40692	16.8123314	5.25E-08	8.70E-07
RORB	-6.36547	10.35263795	0.006456462	0.02539542
BC047605	-6.27905	6.920291918	0.000691085	0.00388775
LBP	-6.25886	9.648470357	0.000812343	0.00445591
ADAMTS4	-6.19165	24.74702381	0.002207851	0.01049271
PROK2	-6.05656	9.663616562	0.002232598	0.0105688
HOXA9	-6.03977	19.57159528	5.47E-16	2.95E-14
PI15	-5.94165	19.00880893	0.002635002	0.01216425
CLDN5	-5.71824	10.53982852	2.48E-10	6.07E-09
TMEM233	-5.67907	20.82264957	0.026133117	0.08146393
TFF3	-5.62271	8.29135374	0.002738144	0.01247377
FAM110D	-5.28716	28.13083213	0.000226906	0.0014875
RGS6	-5.14325	13.82441078	0.000177291	0.00120468
BMX	-5.11986	15.15734693	3.31E-08	5.67E-07
LDLR	-5.0365	20.6688421	1.75E-07	2.51E-06

MT1X	-4.94598	42.37799476	3.48E-10	8.18E-09
AOX1	-4.7587	19.11903281	1.01E-07	1.51E-06
NR4A3	-4.72595	0.294851078	0.009924942	0.03687931
SOX17	-4.71135	13.42897771	1.24E-11	3.67E-10
HOXA10	-4.64858	25.12110005	8.80E-34	3.55E-31
RBPMS2	-4.57446	19.4762695	4.20E-10	9.67E-09
MCF2L	-4.42802	3.186038904	0.002634643	0.01216425
NR4A1	-4.38657	3.695266264	0.00600465	0.02412832
MT2A	-4.38131	23.86477846	5.38E-07	6.85E-06
CCM2L	-4.29128	3.743291761	0.00418471	0.01766634
TUSC5	-4.26888	52.17887205	1.38E-24	1.76E-22
PLIN4	-4.257	15.7998668	0.006036413	0.02417563
CLSTN2	-4.25008	27.40363289	2.96E-06	3.27E-05
DPY19L2	-4.22369	11.27650119	5.63E-06	5.72E-05
C7	-4.17668	5.588163476	0.01354179	0.04773192
SERPINA5	-4.11836	19.96693122	1.71E-05	0.0001524
BRE-AS1	-4.1119	24.53430044	1.34E-21	1.41E-19
ADAMTS1	-4.08496	6.01431113	0.003487817	0.01525683
TSPAN7	-4.06452	10.46124622	4.24E-06	4.42E-05
MEDAG	-4.02678	13.42752618	0.002553318	0.01188013
KANK3	-3.8917	9.043617349	0.00767579	0.02937933
THBS4	-3.88021	27.27968451	4.07E-13	1.49E-11
PCDH17	-3.78562	13.09585625	0.022221256	0.07157552
PHYHIP	-3.77722	28.26483371	0.005972331	0.02403838
EBF2	-3.75141	7.686923132	0.026891861	0.08318595
DGAT2	-3.74582	6.826814059	0.001260319	0.00648662
SLC39A14	-3.71396	1.007899039	0.029921552	0.08991333
Myopodin	-3.66343	43.82118736	8.81E-24	1.07E-21
MUSTN1	-3.65429	48.92013889	3.77E-07	4.85E-06
PTPRQ	-3.64299	13.63114646	1.81E-07	2.58E-06
C9orf47	-3.62157	6.049101262	0.003140082	0.0140145
FBXO16	-3.58903	5.712883738	0.02273195	0.07292916
SSTR1	-3.50912	19.8560608	1.69E-06	1.96E-05
TAL1	-3.48942	58.33994709	2.40E-07	3.24E-06
ZBTB16	-3.48632	5.291132479	0.007078422	0.02735256

FAM198A	-3.46243	30.63808807	5.33E-21	5.16E-19
PRG4	-3.4491	18.9573628	4.51E-10	1.01E-08
G0S2	-3.4349	11.49384138	0.001592875	0.00790804
PPP1R1A	-3.43103	21.8707483	0.024076092	0.07583342
SOX18	-3.39546	11.6394386	0.029319315	0.0885436
SCN3B	-3.39291	34.88705507	9.96E-08	1.51E-06
FBP2	-3.34205	3.200510649	0.001318928	0.00673099
KCNK1	-3.32402	26.46076342	9.50E-18	6.56E-16
ATP1A2	-3.31381	29.0010915	0.000167244	0.00114933
C1QTNF3	-3.29007	6.945108646	0.001187426	0.00616391
GPC3	-3.27342	3.370215311	0.021246796	0.06898792
NNMT	-3.26587	28.17046622	2.33E-10	5.75E-09
HIF3A	-3.20512	21.03174603	0.02854558	0.08685756
MPZL2	-3.20496	9.51155318	7.25E-06	7.19E-05
DGKD	-3.07944	21.03626235	0.030698957	0.0919069
MMRN2	-3.07221	16.58019662	1.59E-07	2.32E-06
C19orf33	-3.07088	17.45394112	0.000416484	0.00248147
GPC6	-3.06675	16.40791854	1.11E-05	0.00010633
GALNT15	-3.02986	18.59920635	3.71E-09	7.74E-08
F5	-3.02819	12.7817197	0.000163332	0.00112886
S1PR3	-3.02476	13.24637275	0.000334071	0.00205147
FAM150B	-3.01748	13.44225838	4.71E-17	3.08E-15
ITIH3	-3.0084	56.41522367	2.41E-05	0.00020891
TPO	down	39.67311305	5.69E-25	8.10E-23
SERTM1	down	12.43678531	0.001309479	0.00669689
TAC1	down	3.534061986	0.030061257	0.09022107

Note: The ones with no fold change is because they do not express at AS sample (FPKM=0) but expressed at healthy control sample. So the log2(fold) is then calculated by log2(0) which is negative inf. The software outputs no value of fold change for these genes but actually they have significant fold change between AS and control.

Supplementary Table 3.KEGG pathways of the DEGs with altered methylation in the promoter regions.

Genes with up-regulated expression and hypo-methylated CpG island in the promoter regions		
KEGG_pathway_Term	P Value	Genes
hsa04380:Osteoclast differentiation	0.000209	PIK3CG, LILRB1, LILRA1, LILRA2, OSCAR, LILRB4, FCGR3A, TYROBP
hsa04620:Toll-like receptor signaling pathway	0.003076	PIK3CG, CCL3, TLR5, CCL5, TLR7, SPP1
hsa05340:Primary immunodeficiency	0.003861	PTPRC, CD8A, IL2RG, CD4
hsa04612:Antigen processing and presentation	0.005512	KLRC4, CD8A, LGMN, CD4, KLRC1
hsa04650:Natural killer cell mediated cytotoxicity	0.005609	PIK3CG, CD48, ITGAL, KLRK1, FCGR3A, TYROBP
hsa04062:Chemokine signaling pathway	0.007769	PIK3CG, CCL3, CXCR4, CCR2, CXCR3, CCL5, WAS
hsa04514:Cell adhesion molecules (CAMs)	0.010505	ITGAL, PTPRC, CD8A, CD2, CD4, CLDN23
hsa04660:T cell receptor signaling pathway	0.015736	PIK3CG, PTPRC, PRKCQ, CD8A, CD4
hsa04611:Platelet activation	0.033535	PIK3CG, TBXAS1, VAMP8, APBB1IP, F2R
hsa04666:Fc gamma R-mediated phagocytosis	0.044159	PIK3CG, PTPRC, INPP5D, WAS
hsa04640:Hematopoietic cell lineage	0.045476	CD8A, CD33, CD2, CD4
hsa05323:Rheumatoid arthritis	0.049542	ITGAL, CCL3, CCL5, ATP6V0D2
**Genes with down-regulated expression and hyper-methylated CpG island in the promoter regions**		
hsa04978:Mineral absorption	0.001196	MT1M, MT2A, ATP1A2, MT1X
hsa04610:Complement and coagulation cascades	0.003842	C7, F5, SERPINA5, C6

Supplementary Table 4.GO annotations of the DEGs with altered methylation in the promoter regions.

**Genes with up-regulated expression and hypo-methylated CpG island in the promoter regions**		
GO_Term	P Value	Genes
GO:0050776~regulation of immune response	1.62E-09	LILRB1, ITGAL, CD96, LAIR1, LILRA1, TRAC, CD8A, CD33, OSCAR, KLRK1, SLAMF7, FCGR3A, KLRC1, TYROBP
GO:0002250~adaptive immune response	2.58E-08	PIK3CG, LILRB1, LAIR1, LAT2, LILRA1, LILRA2, LILRB4, KLRK1, EOMES, CD4, SLAMF7, TRAT1
GO:0006954~inflammatory response	5.36E-08	PIK3CG, ITGAL, CCL3, SCUBE1, LYZ, TLR5, CXCR3, CCL5, TLR7, CCRL2, PRKCQ, P2RX7, KLRG1, CXCR4, CCR2, SPP1, F2R
GO:0006955~immune response	1.24E-06	CCL3, CD8A, GZMA, CYSLTR2, GPR65, TRGV3, CCL5, WAS, IGSF6, CD96, RGS1, CCR2, CD4, IL2RG, TNFSF12-TNFSF13, FCGR3A
GO:0007166~cell surface receptor signaling pathway	1.86E-06	CD101, PTPRC, CD8A, CXCR3, MARCO, IGSF6, P2RX7, DOK2, LILRA1, KLRG1, CD2, CD4, KLRC1
GO:0007165~signal transduction	1.27E-05	ITGAL, KLRK1, RASSF7, APBB1IP, CD48, PDE6B, LPXN, TAGAP, DAPP1, LILRA2, EVI2A, CD4, IL2RG, INPP5D, ARHGAP8, TYROBP, TRAT1, DAPK1, LILRB1, DOK2, RGS1, CD33, LILRB4, SRGAP3, TNFSF12-TNFSF13
GO:0070098~chemokine-mediated signaling pathway	0.00024757	CCRL2, CCL3, CXCR4, CCR2, CXCR3, CCL5
GO:0007155~cell adhesion	0.00029116	PARVG, ITGAL, SLAMF7, CXCR3, CD96, LPXN, CD33, TROAP, CD2, DSC2, CD4, GPNMB, SPP1
GO:0002407~dendritic cell chemotaxis	0.00030454	PIK3CG, CXCR4, CCR2, CCL5
GO:0050850~positive regulation of calcium-mediated signaling	0.00050175	CCL3, CD8A, CD4, TRAT1
GO:0042110~T cell activation	0.0005153	PIK3CG, CD48, CD8A, CD2, WAS
GO:0019722~calcium-mediated signaling	0.00070455	LAT2, CCL3, CXCR4, CXCR3, RCAN3
GO:0050852~T cell receptor signaling pathway	0.00115959	PTPRC, PRKCQ, TRAC, CD4, INPP5D, WAS, TRAT1
GO:0019441~tryptophan catabolic process to kynurenine	0.00127396	KYNU, TDO2, KMO
GO:0042102~positive regulation of T cell proliferation	0.00130139	PTPRC, PRKCQ, CD4, CCL5, SASH3
GO:0006968~cellular defense response	0.00147074	KLRC4, KLRG1, CCR2, TRAT1, TYROBP
GO:0010818~T cell chemotaxis	0.0016898	PIK3CG, CCL3, CXCR3
GO:0006952~defense response	0.00196025	CD48, LILRA1, LILRA2, WAS, SP140

GO:0071222~cellular response to lipopolysaccharide	0.00204893	LILRB1, STAP1, KLRK1, ABCA1, TLR5, NR1H3
GO:0006569~tryptophan catabolic process	0.00216135	KYNU, TDO2, KMO
GO:0045087~innate immune response	0.00224265	PIK3CG, TNFAIP8L2, MARCO, C1QA, KLRG1, KLRK1, LY9, TLR5, TLR7, APOBEC3C, TYROBP
GO:0006935~chemotaxis	0.00285984	CCRL2, CCL3, CXCR4, CCR2, CXCR3, CCL5
GO:0007204~positive regulation of cytosolic calcium ion concentration	0.00427603	PIK3CG, CXCR4, CCR2, CD52, CXCR3, F2R
GO:0006915~apoptotic process	0.00544287	CXCR4, GZMA, GPR65, CD2, BUB1B, BIRC5, INPP5D, CXCR3, TNFSF12-TNFSF13, RASSF7, MELK, DAPK1
GO:0007169~transmembrane receptor protein tyrosine kinase signaling pathway	0.00712668	DOK2, STAP1, CD8A, CD4, TRAT1
GO:0030101~natural killer cell activation	0.0107757	CD2, KLRK1, SLAMF7
GO:0019835~cytolysis	0.01184872	P2RX7, GZMA, LYZ
GO:0070374~positive regulation of ERK1 and ERK2 cascade	0.01280086	CCL3, CYSLTR2, CCL5, GPNMB, ARHGAP8, F2R
GO:0043547~positive regulation of GTPase activity	0.01444209	TAGAP, CCL3, RGS1, ACAP1, SRGAP3, CYTH4, IL2RG, RGS18, CCL5, ARHGAP8, FGD6
GO:0045671~negative regulation of osteoclast differentiation	0.01533383	CCL3, LILRB4, INPP5D
GO:2000473~positive regulation of hematopoietic stem cell migration	0.01577861	PTPRC, CCR2
GO:0019442~tryptophan catabolic process to acetyl-CoA	0.01577861	KYNU, TDO2
GO:0050900~leukocyte migration	0.01611408	CD48, ITGAL, DOK2, CD2, INPP5D
GO:0051928~positive regulation of calcium ion transport	0.01787138	CCL3, CCL5, F2R
GO:0050729~positive regulation of inflammatory response	0.02019967	CCL3, CCR2, CCL5, TLR7
GO:0048873~homeostasis of number of cells within a tissue	0.02198425	P2RX7, SASH3, F2R
GO:0002291~T cell activation via T cell receptor contact with antigen bound to MHC molecule on antigen presenting cell	0.02357501	ITGAL, APBB1IP
GO:0043420~anthranilate metabolic process	0.02357501	KYNU, KMO
GO:0019805~quinolinate biosynthetic process	0.02357501	KYNU, KMO

GO:0007186~G-protein coupled receptor signaling pathway	0.02605438	PIK3CG, CCL3, GPR65, RGS18, ABCA1, CXCR3, CCL5, CCRL2, GPR34, RGS1, CXCR4, CCR2, SUCNR1, F2R
GO:0009968~negative regulation of signal transduction	0.02960931	RGS1, RGS18, INPP5D
GO:0050918~positive chemotaxis	0.03124371	CCL3, CCL5, GPNMB
GO:0034354~'de novo' NAD biosynthetic process from tryptophan	0.0313101	KYNU, KMO
GO:0043308~eosinophil degranulation	0.0313101	CCL3, VAMP8
GO:0030260~entry into host cell	0.0313101	CXCR4, CD4
GO:0045919~positive regulation of cytolysis	0.0313101	LILRB1, P2RX7
GO:0042130~negative regulation of T cell proliferation	0.03461663	LILRB1, GPNMB, VSIG4
GO:2001199~negative regulation of dendritic cell differentiation	0.03898438	LILRB1, TMEM176B
GO:0001915~negative regulation of T cell mediated cytotoxicity	0.03898438	LILRB1, PTPRC
GO:0051209~release of sequestered calcium ion into cytosol	0.04176106	PTPRC, P2RX7, F2R
GO:0051607~defense response to virus	0.04226899	LILRB1, PTPRC, CD8A, TLR7, APOBEC3C
GO:0097191~extrinsic apoptotic signaling pathway	0.04362658	P2RX7, KRT8, TNFSF12-TNFSF13
GO:0060706~cell differentiation involved in embryonic placenta development	0.04659831	KRT8, EOMES
GO:0010887~negative regulation of cholesterol storage	0.04659831	ABCA1, NR1H3
GO:0061737~leukotriene signaling pathway	0.04659831	RGS1, CYSLTR2
**Genes with down-regulated expression and hyper-methylated CpG island in the promoter regions**		
GO:0045926~negative regulation of growth	6.14E-05	MT1M, GPC3, MT2A, MT1X
GO:0001525~angiogenesis	0.00030609	TAL1, PROK2, APOD, HIF3A, SOX18, SOX17, MMRN2
GO:0071294~cellular response to zinc ion	0.0027199	MT1M, MT2A, MT1X
GO:0048866~stem cell fate specification	0.00820156	SOX18, SOX17
GO:0036018~cellular response to	0.00820156	MT2A, MT1X

erythropoietin		
GO:0030522~intracellular receptor signaling pathway	0.01063405	NR4A1, RORB, NR4A3
GO:0003151~outflow tract morphogenesis	0.01532978	CLDN5, SOX18, SOX17
GO:0060214~endocardium formation	0.01633682	SOX18, SOX17
GO:0007204~positive regulation of cytosolic calcium ion concentration	0.01775463	PROK2, S1PR3, AVPR1A, TAC1
GO:0019233~sensory perception of pain	0.01933272	PROK2, SCN3B, TAC1
GO:0060956~endocardial cell differentiation	0.02037975	SOX18, SOX17
GO:0007283~spermatogenesis	0.02095653	PROK2, SSTR1, SERPINA5, HOXA10, HOXA9, SOX17
GO:0043401~steroid hormone mediated signaling pathway	0.02296607	NR4A1, RORB, NR4A3
GO:0071376~cellular response to corticotropin-releasing hormone stimulus	0.02440631	NR4A1, NR4A3
GO:0071638~negative regulation of monocyte chemotactic protein-1 production	0.02440631	APOD, C1QTNF3
GO:0035356~cellular triglyceride homeostasis	0.02841655	DGAT2, C1QTNF3
GO:0061469~regulation of type B pancreatic cell proliferation	0.03241055	NR4A1, NR4A3
GO:0072091~regulation of stem cell proliferation	0.03241055	SOX18, SOX17
GO:0034383~low-density lipoprotein particle clearance	0.03241055	LDLR, DGAT2
GO:0045444~fat cell differentiation	0.03626703	C1QTNF3, NR4A1, NR4A3
GO:0046339~diacylglycerol metabolic process	0.03638837	DGAT2, DGKD
GO:0010038~response to metal ion	0.04035007	MT2A, MT1X
GO:0009952~anterior/posterior pattern specification	0.04282073	HOXA10, HOXA9, ZBTB16
GO:0010867~positive regulation of triglyceride biosynthetic process	0.04429571	LDLR, DGAT2
GO:0045944~positive regulation of transcription from RNA polymerase II promoter	0.04725133	CSF3, TAL1, EBF2, HOXA10, HIF3A, NR4A1, SOX18, SOX17, NR4A3
GO:0045778~positive regulation of ossification	0.04822537	TAC1, ZBTB16

GO:0035815~positive regulation of renal sodium excretion	0.04822537	AVPR1A, TAC1

Supplementary Table 5.Differential expression genes in atheroma plaque *vs*. intact tissue.

**Up-regulated genes in atheroma plaque than in intact tissue**			
Gene.symbol	Adjusted P	logFC	Gene.title
SP140	0.000017	0.734813	SP140 nuclear body protein
OSBPL3	2.36E-05	0.806669	oxysterol binding protein like 3
INPP5D	2.39E-05	0.733255	inositol polyphosphate-5-phosphatase D
CYTH4	2.85E-05	0.876796	cytohesin 4
LRMP	3.69E-05	0.73868	lymphoid restricted membrane protein
RBM47	3.69E-05	0.815586	RNA binding motif protein 47
TYROBP	4.54E-05	0.841439	TYRO protein tyrosine kinase binding protein
CD4	4.75E-05	0.934919	CD4 molecule
APBB1IP	4.93E-05	0.886038	amyloid beta precursor protein binding family B member 1 interacting protein
LAIR1	5.21E-05	0.895547	leukocyte associated immunoglobulin like receptor 1
DCSTAMP	5.35E-05	1.005814	dendrocyte expressed seven transmembrane protein
TBXAS1	5.43E-05	0.767749	thromboxane A synthase 1
ITGAL	5.83E-05	0.763293	integrin subunit alpha L
C1QA	6.76E-05	0.842387	complement C1q A chain
LPXN	7.04E-05	0.694234	leupaxin
TMEM26	7.21E-05	0.462497	transmembrane protein 26
TDO2	7.34E-05	1.150758	tryptophan 2,3-dioxygenase
PIK3CG	7.79E-05	0.723562	phosphatidylinositol-4,5-bisphosphate 3-kinase catalytic subunit gamma
CD33	7.95E-05	0.646166	CD33 molecule
VSIG4	8.12E-05	0.808859	V-set and immunoglobulin domain containing 4
LY9	8.68E-05	0.378471	lymphocyte antigen 9
KMO	9.06E-05	1.124363	kynurenine 3-monooxygenase (kynurenine 3-hydroxylase)
IL2RG	0.000102	0.91399	interleukin 2 receptor subunit gamma
LGMN	0.000103	0.657581	legumain
TNFAIP8L2	0.000106	0.407538	TNF alpha induced protein 8 like 2
FGD6	0.000107	0.48512	FYVE, RhoGEF and PH domain containing 6
TAGAP	0.000115	0.663041	T-cell activation RhoGTPase activating protein
DAPP1	0.000116	0.776533	dual adaptor of phosphotyrosine and 3-

			phosphoinositides 1
ABCC3	0.000125	0.499857	ATP binding cassette subfamily C member 3
CD48	0.00013	0.896888	CD48 molecule
CD52	0.000135	1.263187	CD52 molecule
PARVG	0.000149	0.670897	parvin gamma
SASH3	0.000157	0.495667	SAM and SH3 domain containing 3
SLAMF8	0.000198	0.856962	SLAM family member 8
P2RX7	0.000217	0.714638	purinergic receptor P2X 7
KYNU	0.000267	1.047221	kynureninase
LAT2	0.000278	0.39497	linker for activation of T-cells family member 2
SLAMF7	0.000278	0.944498	SLAM family member 7
LILRB4	0.000282	0.948193	leukocyte immunoglobulin like receptor B4
GMFG	0.000292	0.872371	glia maturation factor gamma
TMEM176B	0.000313	0.598434	transmembrane protein 176B
APOBEC3C	0.00033	0.291823	apolipoprotein B mRNA editing enzyme catalytic subunit 3C
NR1H3	0.000351	0.766277	nuclear receptor subfamily 1 group H member 3
DOK2	0.000358	0.634386	docking protein 2
LILRB1	0.00037	0.695449	leukocyte immunoglobulin like receptor B1
TRAF3IP3	0.000377	0.536955	TRAF3 interacting protein 3
MS4A4A	0.000418	0.938973	membrane spanning 4-domains A4A
DAPK1	0.000418	0.56557	death associated protein kinase 1
PTPRC	0.000441	0.790281	protein tyrosine phosphatase, receptor type C
APOBR	0.00051	0.504868	apolipoprotein B receptor
VAMP8	0.000523	0.850513	vesicle associated membrane protein 8
ATP6V0D2	0.000531	1.42507	ATPase H+ transporting V0 subunit d2
GPR65	0.000533	0.803471	G protein-coupled receptor 65
RAB42	0.00057	0.539136	RAB42, member RAS oncogene family
LYZ	0.000643	0.89793	lysozyme
KCNA3	0.000655	0.69079	potassium voltage-gated channel subfamily A member 3
EVI2A	0.000663	0.696684	ecotropic viral integration site 2A
OSCAR	0.000699	0.301462	osteoclast associated, immunoglobulin-like receptor
ARL11	0.000718	0.341956	ADP ribosylation factor like GTPase 11
WAS	0.00073	0.428687	Wiskott-Aldrich syndrome
MKI67	0.00082	0.628757	marker of proliferation Ki-67
ADAM28	0.000844	0.728816	ADAM metallopeptidase domain 28

DSC2	0.000863	0.718324	desmocollin 2
IKZF3	0.001017	0.504934	IKAROS family zinc finger 3
ABRACL	0.001271	0.406791	ABRA C-terminal like
MS4A6A	0.001566	0.659031	membrane spanning 4-domains A6A
RCAN3	0.001999	0.402779	RCAN family member 3
CCL3	0.002205	0.72473	C-C motif chemokine ligand 3
FCGR3A	0.002209	0.587133	Fc fragment of IgG receptor IIIa
C15orf48	0.002328	0.850664	chromosome 15 open reading frame 48
ARL4C	0.002365	0.539493	ADP ribosylation factor like GTPase 4C
MLPH	0.002474	0.357323	melanophilin
CXCR4	0.002977	0.733738	C-X-C motif chemokine receptor 4
PRKCQ	0.003391	0.29882	protein kinase C theta
GPNMB	0.003558	0.413603	glycoprotein nmb
KLRG1	0.003778	0.545981	killer cell lectin like receptor G1
LILRA1	0.003999	0.408118	leukocyte immunoglobulin like receptor A1
ASF1B	0.004414	0.248729	anti-silencing function 1B histone chaperone
BUB1B	0.004512	0.299599	BUB1 mitotic checkpoint serine/threonine kinase B
TLR5	0.00461	0.39313	toll like receptor 5
GAL3ST4	0.004759	0.178324	galactose-3-O-sulfotransferase 4
MELK	0.005172	0.453615	maternal embryonic leucine zipper kinase
SPP1	0.005223	0.732547	secreted phosphoprotein 1
RGS1	0.005425	1.094815	regulator of G-protein signaling 1
IGSF6	0.005748	0.598643	immunoglobulin superfamily member 6
CCR2	0.006198	0.665934	C-C motif chemokine receptor 2
GAPT	0.006268	0.500738	GRB2-binding adaptor protein, transmembrane
GAS2L3	0.006465	0.600409	growth arrest specific 2 like 3
CCR2	0.006702	0.673273	C-C motif chemokine receptor 2
KIF20A	0.00715	0.319472	kinesin family member 20A
SAMD3	0.007251	0.363712	sterile alpha motif domain containing 3
RUNX3	0.007317	0.221726	runt related transcription factor 3
ABCA1	0.008679	0.618414	ATP binding cassette subfamily A member 1
CD96	0.010228	0.526478	CD96 molecule
MARCO	0.010576	0.756033	macrophage receptor with collagenous structure
CD101	0.010608	0.282858	CD101 molecule
GPR34	0.010613	0.511924	G protein-coupled receptor 34

STAP1	0.010942	0.344782	signal transducing adaptor family member 1
PDE6B	0.011124	0.146415	phosphodiesterase 6B
RGS18	0.011539	0.469008	regulator of G-protein signaling 18
MYBL2	0.011829	0.174273	MYB proto-oncogene like 2
TLR7	0.01282	0.553648	toll like receptor 7
LILRA2	0.018283	0.370121	leukocyte immunoglobulin like receptor A2
CCL5	0.023027	0.495952	C-C motif chemokine ligand 5
CD2	0.02442	0.431276	CD2 molecule
KLRC1	0.028104	0.22484	killer cell lectin like receptor C1
GZMA	0.032827	0.454223	granzyme A
TK1	0.033425	0.15658	thymidine kinase 1
CD8A	0.034795	0.209924	CD8a molecule
APOC1	0.045641	0.698262	apolipoprotein C1
TRAT1	0.049007	0.381661	T cell receptor associated transmembrane adaptor 1
**Down-regulated genes in atheroma plaque than in intact tissue**			
GPC3	1.91E-05	-0.90553	glypican 3
ATP1A2	2.36E-05	-0.85218	ATPase Na+/K+ transporting subunit alpha 2
ADAMTS1	0.000262	-0.62713	ADAM metallopeptidase with thrombospondin type 1 motif 1
RBPMS2	0.000371	-0.28513	RNA binding protein with multiple splicing 2
DPY19L2	0.000958	-0.46136	dpy-19 like 2
GALNT15	0.00138	-0.57328	polypeptide N-acetylgalactosaminyltransferase 15
ZBTB16	0.001851	-0.6847	zinc finger and BTB domain containing 16
MEDAG	0.003072	-0.38058	mesenteric estrogen dependent adipogenesis
AVPR1A	0.008679	-0.23005	arginine vasopressin receptor 1A
RORB	0.033712	-0.12479	RAR related orphan receptor B

Supplementary Table 6. Differential expression genes in advanced *vs.* early atherosclerotic artery tissue.

Up-regulated genes in advanced than in early AS atherosclerotic artery			
Gene.symbol	Adjusted P	logFC	Gene.title
ABCA1	2.59E-04	1.162925	ATP binding cassette subfamily A member 1
ABCA1	7.70E-04	0.999779	ATP binding cassette subfamily A member 1
ABRACL	4.34E-02	0.99401	ABRA C-terminal like
ADAM28	1.45E-03	1.245846	ADAM metallopeptidase domain 28
ADAM28	4.07E-02	0.340491	ADAM metallopeptidase domain 28
APBB1IP	1.34E-03	0.960008	amyloid beta precursor protein binding family B member 1 interacting protein
APOBEC3C	4.54E-02	0.245527	apolipoprotein B mRNA editing enzyme catalytic subunit 3C
APOC1	1.24E-03	0.878254	apolipoprotein C1
APOC1	1.44E-03	1.822423	apolipoprotein C1
ARL11	4.47E-02	0.372961	ADP ribosylation factor like GTPase 11
ARL4C	1.20E-03	0.919834	ADP ribosylation factor like GTPase 4C
ARL4C	1.43E-03	1.883505	ADP ribosylation factor like GTPase 4C
ARL4C	9.46E-03	1.285685	ADP ribosylation factor like GTPase 4C
BUB1B	1.58E-03	0.394727	BUB1 mitotic checkpoint serine/threonine kinase B
C15orf48	1.49E-03	1.364491	chromosome 15 open reading frame 48
C1QA	1.50E-04	1.452946	complement C1q A chain
CCL5	7.58E-03	1.087311	C-C motif chemokine ligand 5
CCL5	7.91E-03	1.075832	C-C motif chemokine ligand 5
CCL5	1.32E-02	0.821746	C-C motif chemokine ligand 5
CCR2	4.14E-03	0.517919	C-C motif chemokine receptor 2
CD2	4.04E-03	0.449125	CD2 molecule
CD33	1.08E-02	0.459837	CD33 molecule
CD4	2.27E-03	0.764285	CD4 molecule
CD48	7.02E-05	1.323418	CD48 molecule
CD52	3.34E-05	1.577455	CD52 molecule
CD52	7.64E-05	1.983144	CD52 molecule
CXCR4	1.92E-03	2.015581	C-X-C motif chemokine receptor 4
CXCR4	2.02E-03	1.734262	C-X-C motif chemokine receptor 4
CXCR4	3.38E-03	1.691841	C-X-C motif chemokine receptor 4
CYTH4	6.55E-06	0.95839	cytohesin 4
DAPK1	4.84E-04	1.23475	death associated protein kinase 1

DAPP1	1.51E-02	0.382939	dual adaptor of phosphotyrosine and 3-phosphoinositides 1
DAPP1	4.45E-02	0.435469	dual adaptor of phosphotyrosine and 3-phosphoinositides 1
DOK2	2.11E-03	0.43852	docking protein 2
DSC2	3.48E-04	0.896625	desmocollin 2
DSC2	6.11E-03	0.309384	desmocollin 2
EVI2A	2.81E-03	1.413796	ecotropic viral integration site 2A
GAS2L3	1.67E-03	0.612177	growth arrest specific 2 like 3
GMFG	2.88E-04	1.05535	glia maturation factor gamma
GPNMB	1.84E-02	0.570354	glycoprotein nmb
GPR34	2.32E-02	1.414389	G protein-coupled receptor 34
GPR65	6.27E-04	0.749496	G protein-coupled receptor 65
GZMA	1.76E-02	0.61803	granzyme A
IGSF6	2.52E-04	1.079022	immunoglobulin superfamily member 6
IL2RG	6.68E-04	0.905319	interleukin 2 receptor subunit gamma
INPP5D	6.12E-04	0.870383	inositol polyphosphate-5-phosphatase D
INPP5D	2.62E-02	0.201731	inositol polyphosphate-5-phosphatase D
ITGAL	2.63E-04	0.887136	integrin subunit alpha L
ITGAL	3.65E-04	0.4475	integrin subunit alpha L
KIF20A	8.39E-03	0.449123	kinesin family member 20A
KMO	3.06E-04	0.931282	kynurenine 3-monooxygenase (kynurenine 3-hydroxylase)
KMO	3.88E-03	0.910496	kynurenine 3-monooxygenase (kynurenine 3-hydroxylase)
KYNU	7.64E-05	2.036197	kynureninase
KYNU	3.11E-03	0.435811	kynureninase
KYNU	4.12E-03	0.743324	kynureninase
LAIR1	4.98E-05	0.548483	leukocyte associated immunoglobulin like receptor 1
LAIR1	1.63E-04	1.456417	leukocyte associated immunoglobulin like receptor 1
LAT2	1.35E-04	0.851482	linker for activation of T-cells family member 2
LGMN	2.26E-03	0.998596	legumain
LILRA2	5.44E-03	0.513172	leukocyte immunoglobulin like receptor A2
LILRA2	2.11E-02	0.284086	leukocyte immunoglobulin like receptor A2
LILRB1	4.31E-04	0.696875	leukocyte immunoglobulin like receptor B1
LILRB1	5.11E-04	0.688577	leukocyte immunoglobulin like receptor B1
LILRB4	1.18E-02	0.846391	leukocyte immunoglobulin like receptor B4
LPXN	2.43E-03	0.874111	leupaxin
LRMP	4.05E-03	0.465468	lymphoid restricted membrane protein

LRMP	9.97E-03	0.542583	lymphoid restricted membrane protein
LY9	4.67E-02	0.27122	lymphocyte antigen 9
MARCO	8.39E-04	1.201199	macrophage receptor with collagenous structure
MELK	1.44E-03	0.5166	maternal embryonic leucine zipper kinase
MKI67	4.59E-03	0.446221	marker of proliferation Ki-67
MLPH	7.08E-03	0.710845	melanophilin
MS4A4A	3.05E-04	1.006839	membrane spanning 4-domains A4A
MS4A4A	1.00E-03	1.761363	membrane spanning 4-domains A4A
MS4A4A	5.40E-03	2.004715	membrane spanning 4-domains A4A
MS4A6A	5.69E-04	1.235292	membrane spanning 4-domains A6A
MS4A6A	1.44E-03	1.374	membrane spanning 4-domains A6A
MS4A6A	1.80E-03	1.385346	membrane spanning 4-domains A6A
MS4A6A	1.97E-03	0.827928	membrane spanning 4-domains A6A
MS4A6A	1.99E-03	1.386866	membrane spanning 4-domains A6A
MS4A6A	2.24E-02	1.083691	membrane spanning 4-domains A6A
NR1H3	7.31E-03	0.609153	nuclear receptor subfamily 1 group H member 3
OSBPL3	2.42E-04	0.954952	oxysterol binding protein like 3
OSBPL3	3.38E-02	0.462119	oxysterol binding protein like 3
OSCAR	1.87E-02	0.390103	osteoclast associated, immunoglobulin-like receptor
P2RX7	3.02E-05	0.859049	purinergic receptor P2X 7
P2RX7	2.61E-04	1.700264	purinergic receptor P2X 7
PARVG	5.47E-05	0.90065	parvin gamma
PARVG	7.11E-05	0.675143	parvin gamma
PIK3CG	4.45E-04	1.102316	phosphatidylinositol-4,5-bisphosphate 3-kinase catalytic subunit gamma
PTPRC	5.60E-04	1.559453	protein tyrosine phosphatase, receptor type C
PTPRC	2.41E-03	1.4814	protein tyrosine phosphatase, receptor type C
PTPRC	5.95E-03	1.742707	protein tyrosine phosphatase, receptor type C
RAB42	2.82E-03	0.369534	RAB42, member RAS oncogene family
RBM47	6.92E-05	0.946652	RNA binding motif protein 47
RBM47	1.28E-04	1.360641	RNA binding motif protein 47
RGS1	2.66E-02	1.485072	regulator of G-protein signaling 1
RGS1	2.91E-02	1.697574	regulator of G-protein signaling 1
RUNX3	1.37E-04	0.699472	runt related transcription factor 3
RUNX3	5.00E-03	0.363502	runt related transcription factor 3
SASH3	4.30E-05	0.91804	SAM and SH3 domain containing 3

SLAMF7	9.19E-04	0.720952	SLAM family member 7
SLAMF7	1.70E-03	0.805796	SLAM family member 7
SLAMF7	6.11E-03	0.321702	SLAM family member 7
SLAMF8	1.90E-05	2.072546	SLAM family member 8
SLAMF8	7.11E-05	1.17073	SLAM family member 8
SP140	4.88E-02	0.266032	SP140 nuclear body protein
SPP1	4.77E-04	3.107965	secreted phosphoprotein 1
SPP1	8.19E-03	1.661208	secreted phosphoprotein 1
TAGAP	5.18E-03	0.872911	T-cell activation RhoGTPase activating protein
TBXAS1	2.91E-03	0.686809	thromboxane A synthase 1
TBXAS1	3.05E-02	0.328366	thromboxane A synthase 1
TLR5	3.14E-03	0.821098	toll like receptor 5
TLR7	8.11E-03	0.651409	toll like receptor 7
TMEM176B	2.15E-03	1.360095	transmembrane protein 176B
TNFAIP8L2	1.62E-02	0.277846	TNF alpha induced protein 8 like 2
TRAF3IP3	7.41E-04	0.586792	TRAF3 interacting protein 3
TYROBP	1.84E-04	1.697715	TYRO protein tyrosine kinase binding protein
VAMP8	2.38E-05	1.825385	vesicle associated membrane protein 8
VSIG4	7.76E-04	1.431208	V-set and immunoglobulin domain containing 4
WAS	1.13E-03	0.378008	Wiskott-Aldrich syndrome
WAS	2.45E-02	0.330596	Wiskott-Aldrich syndrome
**Down-regulated genes in advanced than in early AS atherosclerotic artery**			
ATP1A2	1.28E-04	-1.83392	ATPase Na+/K+ transporting subunit alpha 2
ATP1A2	1.32E-03	-1.46999	ATPase Na+/K+ transporting subunit alpha 2

Note: Each of the duplicate gene symbols represents individual probe for the same gene
